# Abnormal Expression and Prognosis Value of COG Complex Members in Kidney Renal Clear Cell Carcinoma (KIRC)

**DOI:** 10.1155/2021/4570235

**Published:** 2021-09-08

**Authors:** Yanjun Zhang, Hui Lai, Bin Tang

**Affiliations:** ^1^School of Pharmacy, Southwest Medical University, Luzhou, Sichuan 646000, China; ^2^Basic Medical College, Southwest Medical University, Luzhou, Sichuan, China; ^3^Department of Pharmacy, The Affiliated T.C.M. Hospital of Southwest Medical University, Luzhou 646000, China; ^4^Key Lab of Process Analysis and Control of Sichuan Universities, Yibin University, Yibin, China

## Abstract

Kidney renal clear cell carcinoma (KIRC) is the most aggressive subtype of kidney tumours with poor prognosis as well as the increasing incidence rate in worldwide. The conserved oligomeric Golgi (COG) complex is an eight-subunit (Cog1-8) peripheral Golgi protein that controls membrane trafficking and protein glycosylation and plays vital roles in human disease including cancers. Therefore, to uncover the prognostic value of COG complex in KIRC, a series of databases, including UALCAN database, GEPIA database, and Kaplan-Meier plotter, were used to analyse the mRNA expression of COG complex subunits and their prognostic values in patients with KIRC in this study. Compared with normal counterparts, mRNA expression of six COG complex subunits was significantly downregulated in KIRC tissue in UALCAN database, while COG4 mRNA expression was significantly upregulated in KIRC tissue. Moreover, the survival analysis indicated that all members of COG complex subunits were closely related with the prognosis of KIRC patients, while COG1 and COG4 were significantly associated with unfavourable overall survival (OS), the rest of COG complex subunits were importantly correlated with favourable OS. Simultaneously, higher mRNA expression of COG3, COG6, and COG8 exhibits better progression-free survival (PFS) and disease-free survival (DFS) in KIRC patients. These results identified that COG complex subunits, especially COG3, COG6, and COG8, are potential prognostic biomarkers of KIRC patients and may offer effective and new strategies for more accurately diagnosing the patients with KIRC in advance.

## 1. Introduction

Kidney renal clear cell carcinoma (KIRC) accounting for 80-90% of patients with renal cell carcinoma (RCC), one of the most lethal malignant urinary tumours, has poor prognosis [1, 2]. Chemotherapy and radiotherapy are poorly effective for patients with KIRC due to the high resistance of KIRC, leading to the higher mortality rate [3]. Although great progress has been made in the therapeutic treatment and the mechanism of recurrence and metastasis, the mortality rate and the diagnose rate are still far from being ideal. At present, the progression of KIRC cannot be also accurately predicted by clinical characteristics or common molecular biomarkers. Therefore, novel useful biomarkers are needed to be identified for early diagnosis, prevention, and personalized treatment.

The conserved oligomeric Golgi (COG) complex is an important evolutionally conserved multisubunit tethering complex at the Golgi apparatus [4, 5]. COG complex plays a crucial role in endosome to Golgi transport, intra-Golgi retrograde vesicle trafficking, and Golgi homeostasis [6, 7]. In addition, COG complex is also essential for proper localization of Golgi glycosylation machinery [8]. COG complex has eight heteromeric subunits that subdivided into two lobes named lobe A (COG1-4) and lobe B (COG5-8) [ 5], and COG1 and COG8 form the major bridging interaction between the two subcomplexes. Accumulating studies have shown that COG complex regulates the intra-Golgi localization of glycosylation enzymes to control N-and O-linked glycosylation of proteins that can modify the behavior of tumour cells and is associated with tumour grades and prognosis [9–11]. In addition, dysfunction of the COG complex can affect the separation of glycosyltransferases from anterograde cargo molecules to interfere normal protein glycosylation [11, 12]. Although the role of COG complex in cancer is not reported more, the abnormal expression of COG complex can affect tumour invasion and metastasis by regulating the glycosylation of protein. Therefore, understanding of the regulation and molecular function of COG complex may identify potential targets for the diagnosis and treatment of KIRC.

In the present research, to investigate the potential prognostic value of COG complex in KIRC patients, we comprehensively analysed the mRNA expression of individual COG subunits in KIRC by mining the databases and then explored the prognostic roles of these genes in KIRC.

## 2. Materials and Methods

### 2.1. UALCAN Database

UALCAN is a web resource which provides a comprehensive cancer transcriptome data (http://ualcan.path.uab.edu/) [13]. The whole COG complex family member expression at mRNA and protein level in KIRC tissues and in corresponding normal gastric tissues was assessed by using UALCAN database. In addition, the UALCAN database was also used to check the relationship between individual COG family member in different clinicopathological features for KIRC patients.

### 2.2. Survival Analysis

Overall survival analysis of KIRC patients with different expression levels of COG isoforms was performed through the Kaplan–Meier plotter (http://kmplot.com/analysis/) [14], a database containing gene expression data and clinical data. Then, the progression-free survival (PFS) of KIRC patients was performed by UCSC Xena browse (http://xenabrowser.net/) [15], where it contains the data from the Cancer Genome Atlas (TCGA). In addition, the prognostic significance depended on disease-free survival (DFS) in KIRC patients with different expression levels of COG isoforms were performed through Gene Expression Profiling Interactive Analysis (GEPIA, http://gepia2.cancer-pku.cn/#survival) [16], which contains TCGA and GTEX' RNA sequencing expression data and provides differential gene expression analysis and survival analysis. A *p* value below 0.05 was considered significant.

## 3. Results

### 3.1. Transcriptional Levels of COG Complex in Patients with KIRC

**Figure 1:**
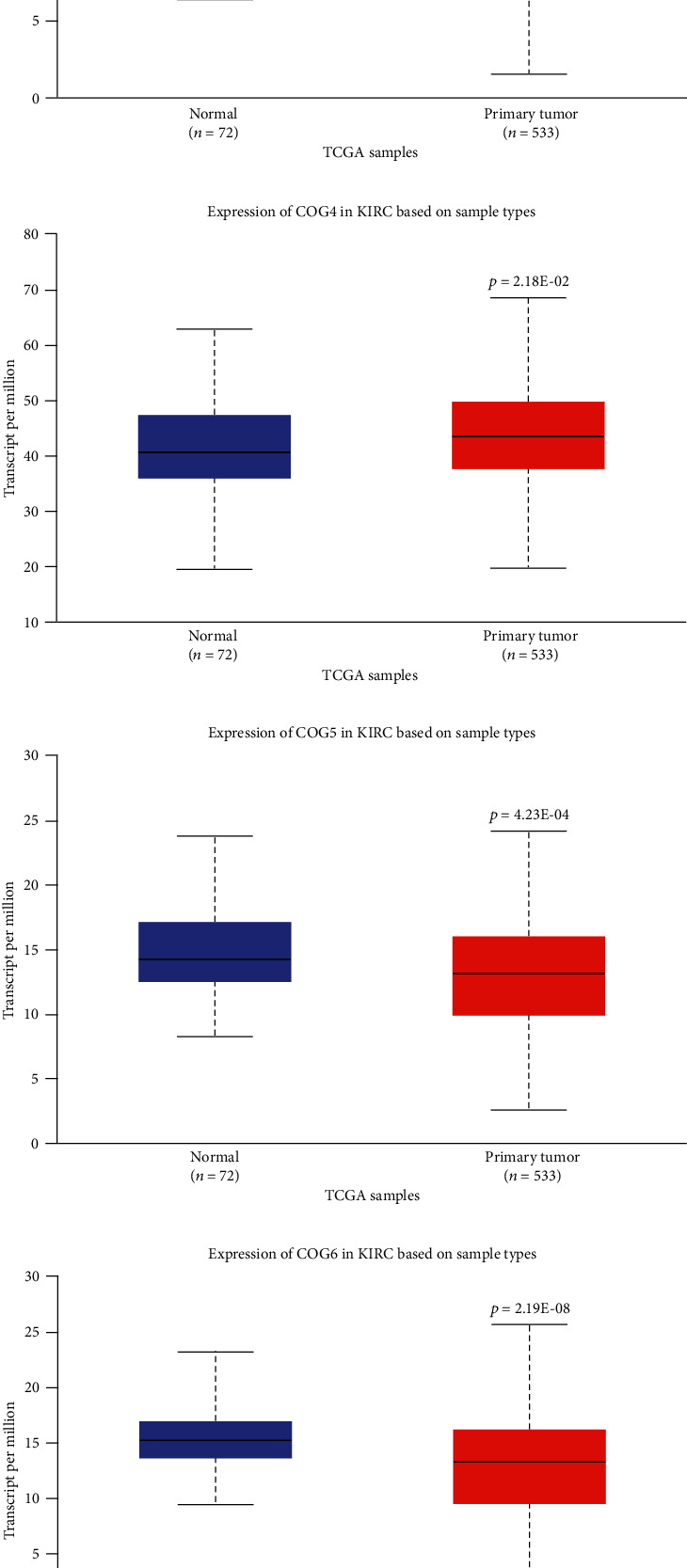
mRNA expression of COG complex members varied in primary tumour and in corresponding normal tissues in KIRC patients using UALCAN database. (a)–(h) mRNA expressions of all COG family members were analysed in KIRC tumour tissues compared to normal tissues.

Eight members of COGs complex have been identified in mammalian cells. We compared the transcriptional levels of 8 COG complex members in KIRC and corresponding normal tissues by UALCAN databases (Figure 1). The results showed that the mRNA expression levels of COG2, COG3, COG5, COG6, COG7, and COG8 were significantly downregulated in KIRC (*p* < 0.05), while only COG4 mRNA expression was significantly increased in patients with KIRC (*p* = 0.0218).

### 3.2. Prognostic Values of COG Complex in KIRC Patients

**Figure 2:**
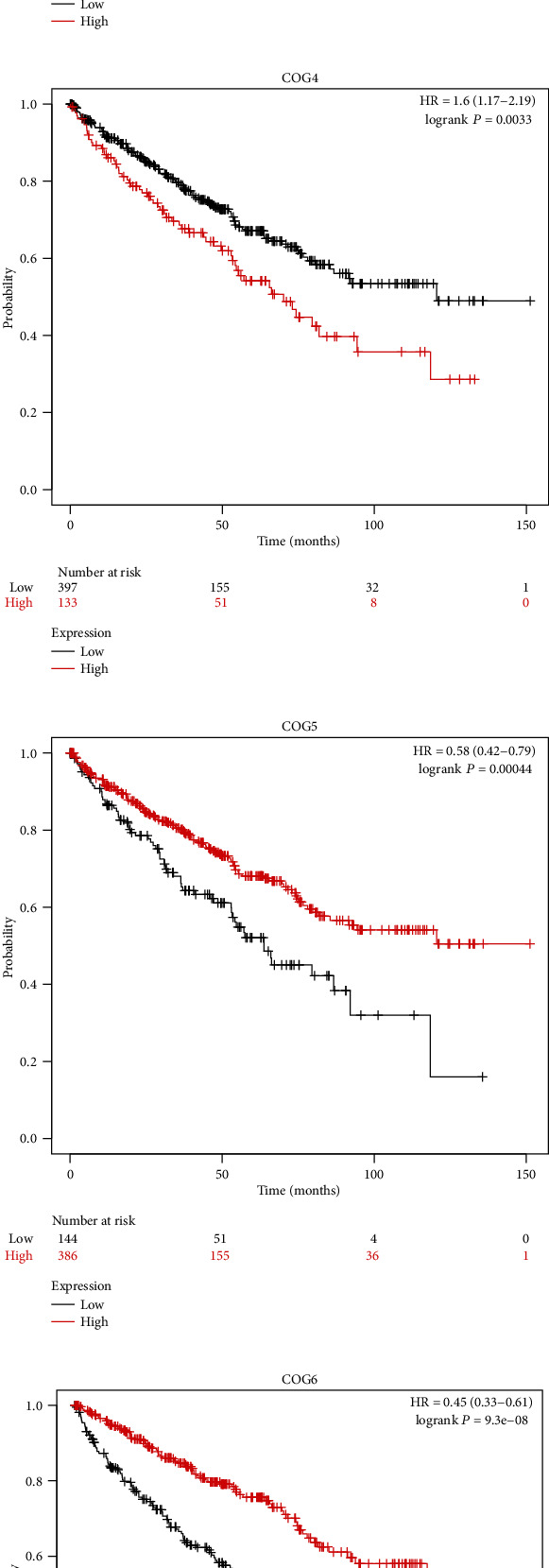
Prognostic value of COG complex related with overall survival in KIRC patients using Kaplan-Meier plotter.

**Figure 3:**
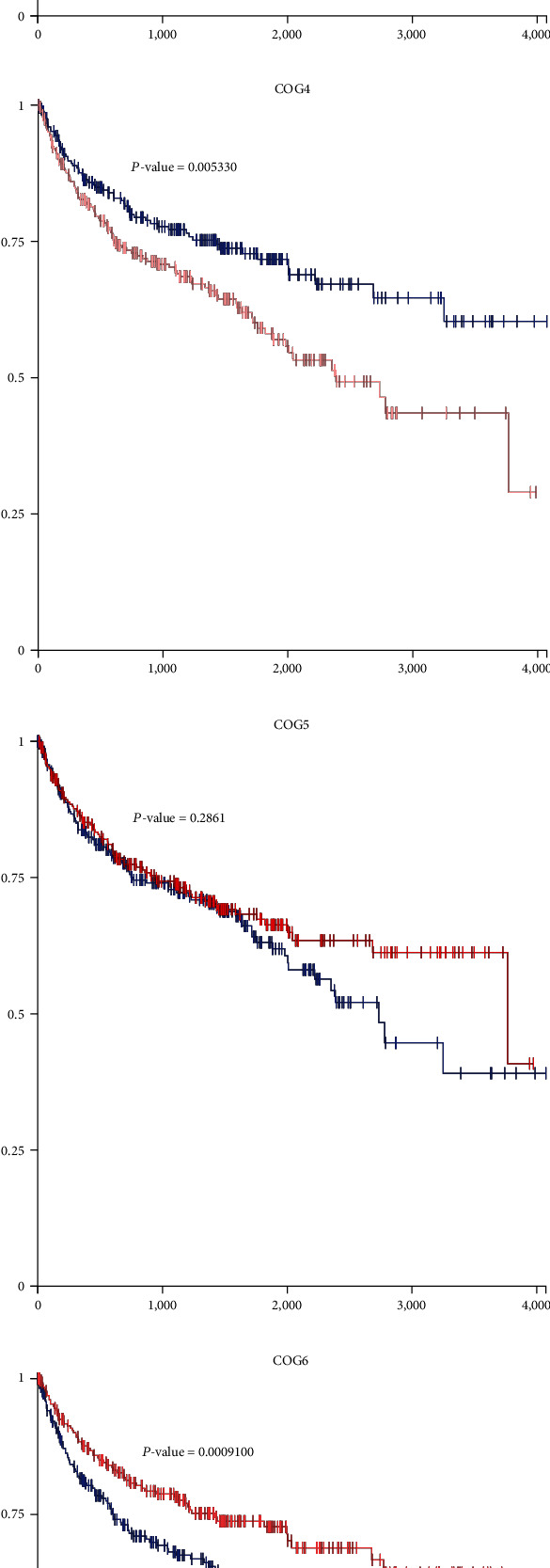
The relationship between COG gene expression levels and patient outcomes in progression-free survival (PFS) of KIRC patients.

**Figure 4:**
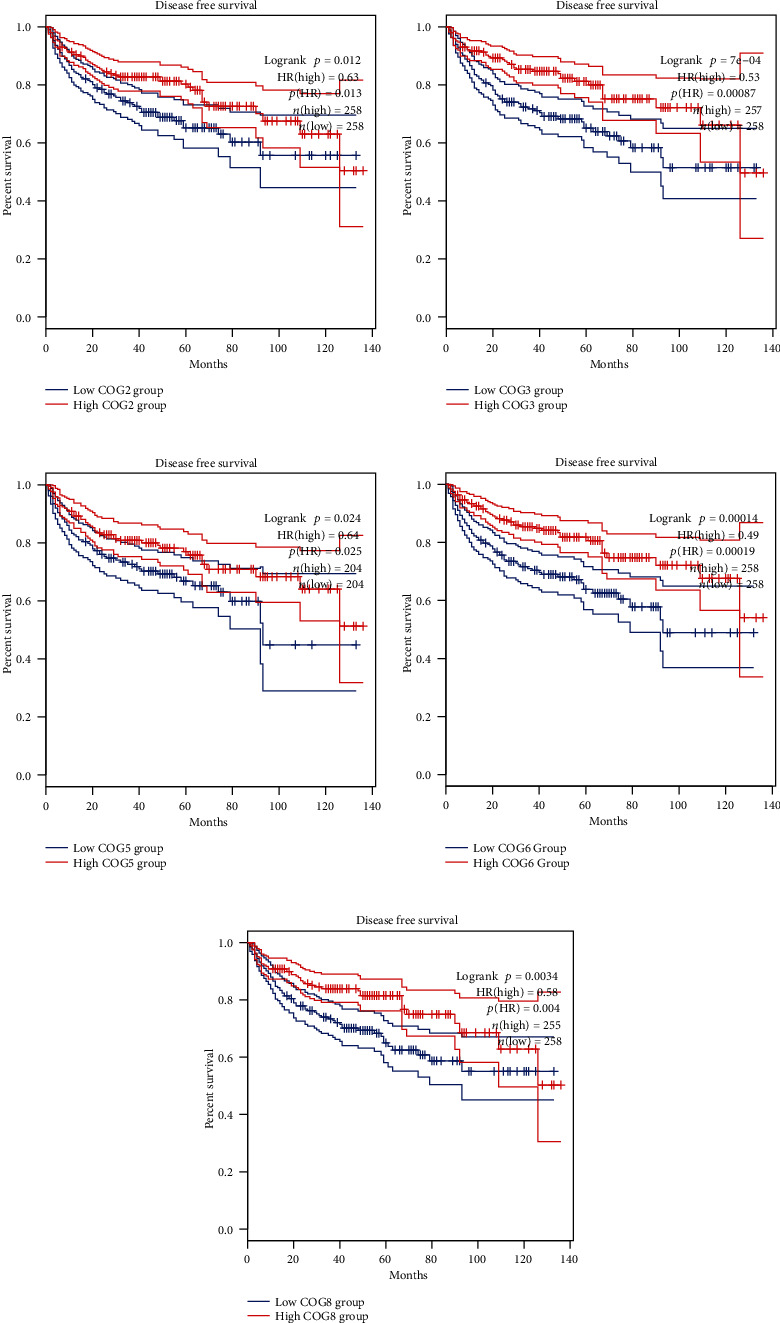
The relationship between COG genes expression levels and patient outcomes in disease-free survival (DFS) of KIRC patients.

In order to assess the prognostic values of COG complex in KIRC, we studied the relationship between COG complex levels and patient outcomes, including overall survival (OS), progression-free survival (PFS), and disease-free survival (DFS) thought using difference online analysis methods. First, Kaplan–Meier plotter analysis showed that among 8 individual COG genes, COG2, 3, 5, 6, 7, and COG8 mRNA high expression was correlated with significantly better OS for all GC patients while high mRNA expression of COG1 and COG4 was significantly associated with a poor prognosis (Figure 2). In addition, progression-free survival (PFS) as the time from the start of treatment to the date of disease progression or death has become a commonly used outcome to assess the efficacy of cancer drugs. To evaluate the association between PFS and health-related quality of life in KIRC patients, we further assessed the correlation between KIRC patients PFS and COG complex gene expression, shown by Kaplan-Meier curves (Figure 3). Moreover, we investigated the expression of COG complex genes and its association with disease-free survival of KIRC patients. Kaplan-Meier showed COG2, 3, 5, 6, and 8 were associated with disease-free survival of patients with KIRC for patients with lower mRNA expression tend to have shorter disease-free survival (Figure 4).

### 3.3. Prognostic Values of COG in KIRC Patients with Different Clinicopathological Features

**Figure 5:**
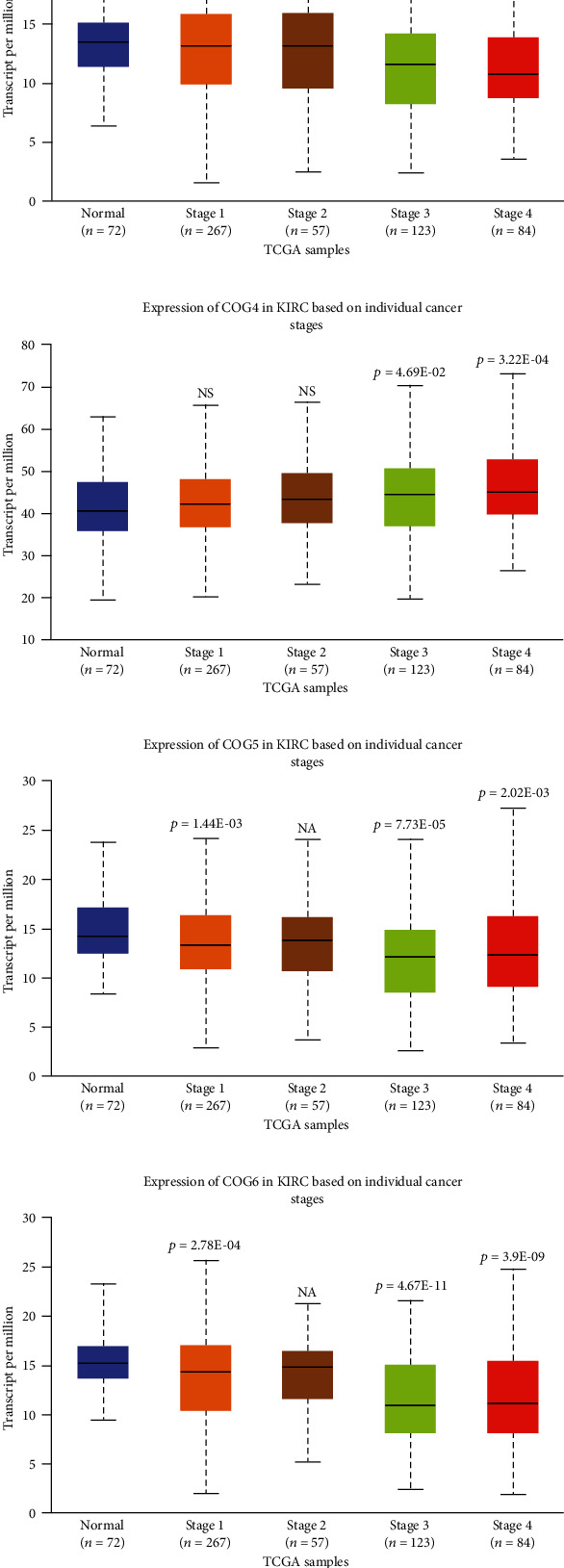
The relationship between COG complex members mRNA expression and tumour stages of KIRC patients in the UALCAN database. (a)–(h) Boxplot showing relative expressions of COG family members in normal individuals or KIRC patients with stage 1, 2, 3, or 4 tumours.

**Figure 6:**
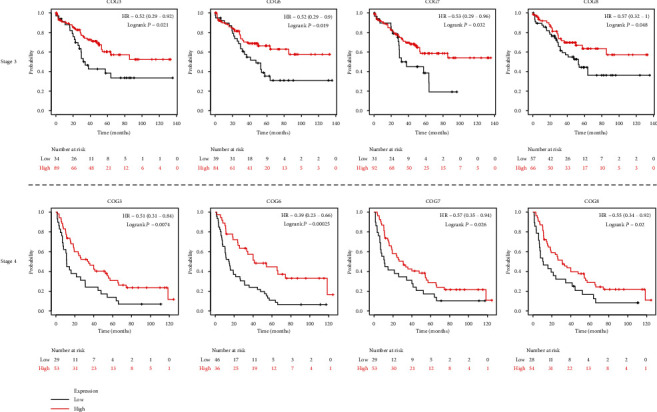
mRNA expressions of individual COG were significantly correlated with patients' individual cancer stage 3 and stage 4 by using Kaplan-Meier plotter.

**Figure 7:**
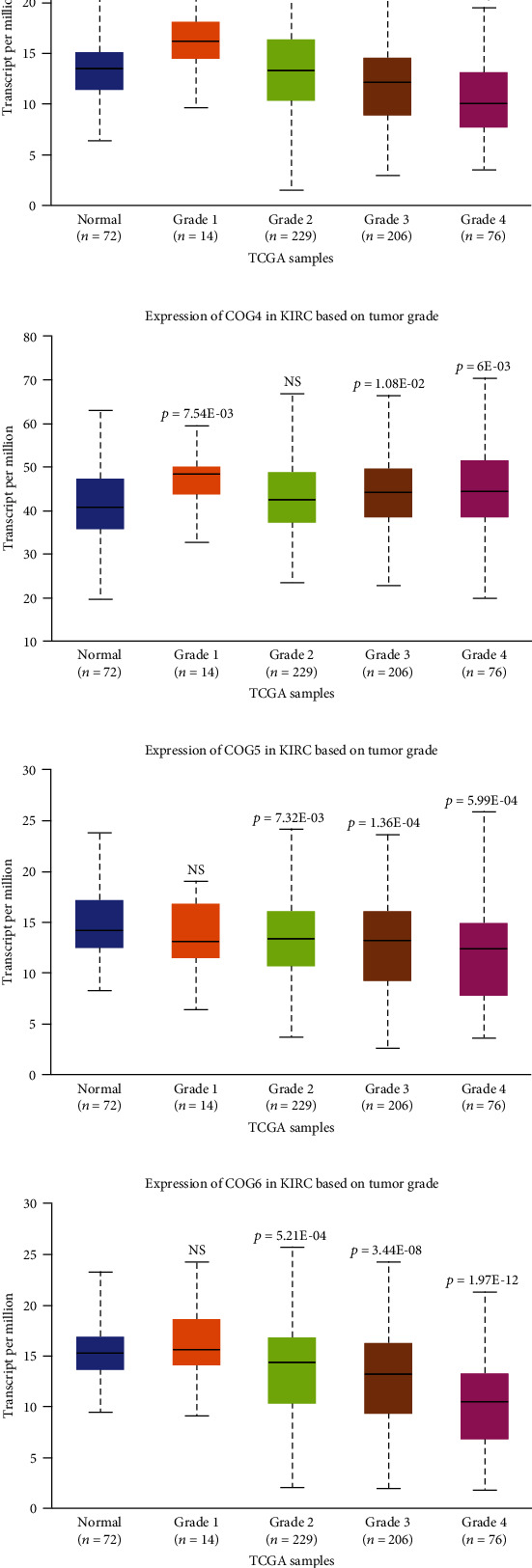
The relationship between COG complex members mRNA expression and tumour grade of KIRC patients in the UALCAN database. (a)–(h) mRNA expression of individual COGs was significantly correlated with tumour grades in KIRC patients.

The relation of COG with other clinicopathological features of KIRC patients, including individual cancer stages, and pathological grade was analysed. The results showed that COG isoform expression was significantly correlated with individual cancer stages of KIRC patients (Figure 5). In addition, Kaplan–Meier plotter analysis showed that lower combinatory mRNA expressions of some individual COG genes including COG 3, 6, 7, and 8 were associated with poorer overall survival (OS) in KIRC patients with stage 3 and stage 4 (Figure 6). Similarly, high mRNA expression of 5 of all COG isoforms (COG2, COG3, COG5, COG6, COG7, and COG8) was also correlated with favourable OS of KIRC patients with tumour grade 2, grade 3, and grade 4, while patients with low mRNA expression of COG4 showed higher survival rate (Figure 7). mRNA expression levels of COG subtypes depended on the tumour grade, especially COG4, COG5, COG6, COG7, and COG8. The highest mRNA expression of COG2 was found in tumour grade 2, and the mRNA expression of COG3 was only associated with grade 3. Taken together, mRNA expressions of COGs family members were significantly linked with clinicopathological features in KIRC patients.

## 4. Discussion

Although novel diagnostic methods were developed to improve the diagnostic efficiency of KIRC, the patients with KIRC still showed the poor prognosis due to the limited biomarkers. Therefore, in order to find new biomarkers for early detection and prognosis, we analysed the expression and clinical values of COGs in KIRC patients by data mining. Our results demonstrate that the mRNA of individual COGs was significantly lower expressed except COG4 whose mRNA expression is significantly increased compared with normal tissue. Moreover, the expression of COG genes is also correlated with stage and grade or KIRC patients. In addition, results from our study showed higher COG3, COG6, and COG8 expression associated with favourable survival, indicating that COG3, COG6, and COG8 may be statistically significant prognostic biomarkers.

Glycosylation is important for tumour progression and metastasis [17]. It has been well documented that aberrant glycosylation contributes to tumorigenesis in multiple cancer types [18–20]. In addition, aberrant glycosylation increased cancer stem cell ability for tumour proliferation [21] and also could weakened immune checkpoint blockade against cancer cells [22, 23]. Simultaneously, accumulating data showed that protein glycosylation and trafficking were regulated by the conserved oligomeric Golgi (COG) complex [24]. COG complex depletions resulted in the resident Golgi glycosyltransferases/glycosidases to be mislocalized or degraded, although Golgi glycosyltransferases are mostly stable. Acute depletion of COG complex subunits caused not only the mislocalization of MAN2A1, MGAT1, B4GALT1, and ST6GAL1 but also altered the stability and/or glycosylation modifications of these proteins [8]. MAN2A1 was reported to involve in glycosylation pattern in cancer cells, and its knockout strengthened the PD-L1 blockade therapy and immune response against cancer cells [22]. N-acetylglucosaminyltransferases (MGAT1) regulated the tumour growth and invasion [25–27]. In addition, the sialyltransferase ST6GAL1 regulated the abnormal glycosylation of some tumour suppressor genes to promote tumourigenesis [28, 29], suggesting that COG complex acts as upstream regulators of these genes to regulate their function in tumours. Moreover, glycosylation and trafficking defects in fibroblasts isolated from COG-deficient human patients have been found through several experimental approaches, suggesting that COG complex plays a key role in tumour metastasis by regulating protein glycosylation. Therefore, COG complex has the potentiality to be mined as cancer biomarkers. The potential mechanism, function, and prognostic value of COG complex in KIRC or other cancer types were needed to be further illustrated for KIRC therapy.

## 5. Conclusion

This study evaluated the prognostic values of COG complex genes in KIRC patients by mining the online database with bioinformatic analysis. Our results suggested that high mRNA expression levels of COG2, COG3, COG5, COG6, COG7, and COG8 were associated with better OS, while only COG4 was significantly related with worse OS in KIRC patients. Moreover, further analysis with PFS and DFS of patients showed that high expression of COG3, COG6, and COG8 was closely correlated with favourable survival. Besides, the analysis between COG genes expression and clinicopathological characteristics in KIRC indicated that COG genes are promising prognostic biomarkers in KIRC patients and may offer novel targets for KIRC therapy. But the mechanism of COG genes as tumour-related protein in KIRC is still insufficient. More researches need to be further elucidated.

## Data Availability

No data were used to support this study.
